# Fault diagnosis method of submersible screw pump based on random forest

**DOI:** 10.1371/journal.pone.0242458

**Published:** 2020-11-16

**Authors:** Minzheng Jiang, Tiancai Cheng, Kangxing Dong, Shufan Xu, Yulong Geng

**Affiliations:** 1 School of Mechanics Science & Engineering, Northeast Petroleum University, Daqing, Heilongjiang, China; 2 The Second Oil Production Plant of Daqing Oilfield Co., Ltd., Daqing, Heilongjiang, China; 3 Daqing Oilfield Construction Group Co., Ltd., Daqing, Heilongjiang, China; University of Bradford, UNITED KINGDOM

## Abstract

The difficulty in directly determining the failure mode of the submersible screw pump will shorten the life of the system and the normal production of the oil well. This thesis aims to identify the fault forms of submersible screw pump accurately and efficiently, and proposes a fault diagnosis method of the submersible screw pump based on random forest. HDFS storage system and MapReduce processing system are established based on Hadoop big data processing platform; Furthermore, the Bagging algorithm is used to collect the training set data. Also, this thesis adopts the CART method to establish the sample library and the decision trees for a random forest model. Six continuous variables, four categorical variables and fault categories of submersible screw pump oil production system are used for training the decision trees. As several decision trees constitute a random forest model, the parameters to be tested are input into the random forest models, and various types of decision trees are used to determine the failure category in the submersible screw pump. It has been verified that the accuracy rate of fault diagnosis is 92.86%. This thesis can provide some meaningful guidance for timely detection of the causes of downhole unit failures, reducing oil well production losses, and accelerating the promotion and application of submersible screw pumps in oil fields.

## Introduction

At present, there are more than 170,000 pumping wells as part of PetroChina, and pumping-unit lifting technology has always occupied the dominant position of artificial lifting [1]. With the increase in the water content of the produced fluid, a new rod pump oil recovery technology-the same well injection and production technology began to be applied [2–4]. However, with the emergence of complex well types and oil production conditions, serious problems such as eccentric wear of pipe and rod caused by rod pump oil production systems are still unavoidable. As a rodless pumping equipment, submersible screw pump is applicable to special situations, such as heavy oil, high sand concentration, places with wax or gas, inclined well or horizontal well. Compared with rod pump, submersible screw pump can avoid the transmission loss caused by the expansion and distortion of sucker rod and improves the efficiency; at the same time, it eliminates the eccentric wear of pipe and rod, reduces the chance of rupture and breaking off, and prolongs the pump inspection cycle [5]. Therefore, submersible screw pump as a new rodless lifting technology is very promising.

However, the main components of the submersible screw pump are all placed underground, such as submersible motor, flexible shaft and screw pump, so manual observation can hardly identify all the problems [6]. Therefore, some failures of submersible screw pumps cannot be discovered in time, and will continue to operate. With the passage of time, the oil wells that only need simple maintenance may have more failures and cause greater economic losses. At present, the commonly used fault diagnosis methods include polished rod force method, electrical parameter method, current method, voltage holding method and neural network-based diagnosis method. The first four methods can only be adopted by experienced workers or experts according to the changes of current, power and other parameters and the working characteristics of screw pump on site. These four methods can hardly be used skillfully or supervised and accuracy rate is relatively low [7]. In order to dispose some faults of screw pump wells widely applied in the oil field exploitation and improve the veracity of fault diagnosis and the capablility of multi-fault diagnosis, Li established an intelligent integrated fault diagnosis expert system based on the fuzzy neural network, and the construction of the system was given [8]. Xue proposed a fault diagnosis method for screw pump wells based on BP neural network and expert system [9]. The fault diagnosis method based on neural network generally analyzes faults based on the active power parameters of submersible screw pump. The diagnosis accuracy is relatively low, and the fault category of submersible screw pump cannot be identified accurately. Random forest algorithm uses machine learning for data mining, which is a method based on statistical analysis and is able to mine information and discover knowledge without clear assumptions [10].

The fault information of submersible screw pump is contained in various working parameters, including both continuous variables and classified variables. The random forest algorithm can deal with both continuous variables and classified variables at the same time, establish a decision tree and form a random forest to assist in decision-making. Therefore, based on a large amount of data generated in the production process and MapReduce parallel processing system, this paper establishes HDFS distributed storage system and Hadoop big data processing platform for diagnosed faults in submersible screw pump.

## Working principle and fault types of submersible screw pump

### Structure and working principles of submersible screw pump

**Fig 1:**
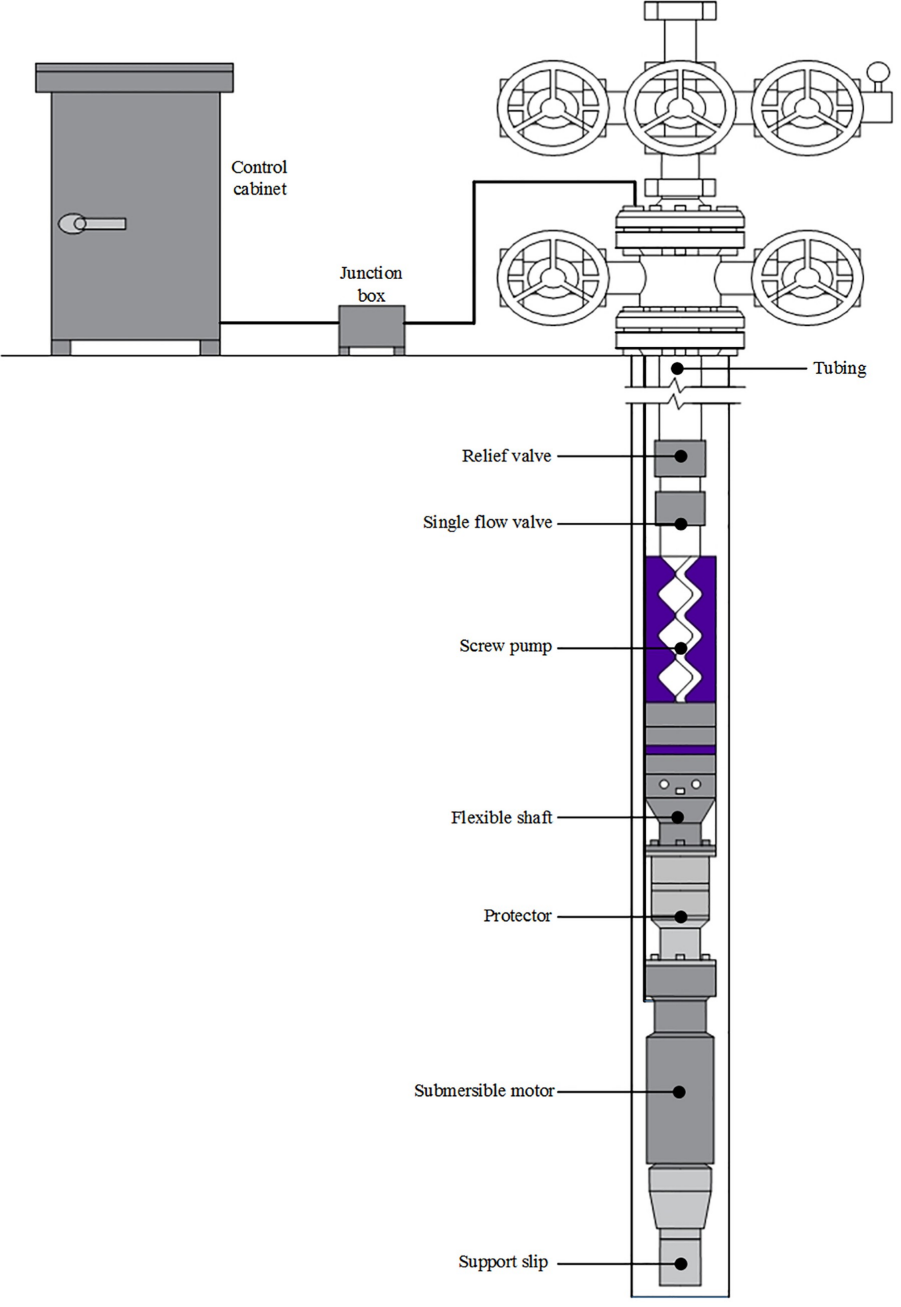
Schematic diagram of production system of submersible screw pump.

As shown in Fig 1, the production system of submersible screw pump is mainly composed of two parts: ground device, including transformer, control cabinet, frequency converter, and junction box and downhole device, like submersible motor, flexible coupling, protector, screw pump, submersible cable, drain valve, and single flow valve. The power from the ground power supply is transmitted to the transformer, control cabinet and junction box first, and then the submersible motor through the submersible cable. Under the action of the protector, the submersible motor propels the flexible shaft to drive the screw pump to rotate at a low speed. After being pressurized by the screw pump, the oil is lifted to the ground through the oil pipeline [11].

### Fault types of submersible screw pump

The failure of the surface-driven screw pump oil production system is mainly divided into two categories, namely, surface fault and downhole fault. Among them, ground faults include abnormal power supply and reducer failure; while downhole faults include broken sucker rod, eccentric wear of sucker rod, tubing trip, pump empty pumping, pump jamming, pump leakage, string leakage, and tubing wax deposition [12]. Compared with the surface-driven screw pump, the submersible screw pump is not equipped with the sucker rod and reducer, and puts the motor upside down in the underground. The screw is connected to the motor with a flexible shaft. The fault of the submersible screw pump system can be divided into seven types: pump emptying, wax blockage, pump leakage, flexible shaft fault, oil pipe leakage, abnormal power supply and pump jamming.

## Big data processing platform and algorithm

### Hadoop ecosystem

**Fig 2:**
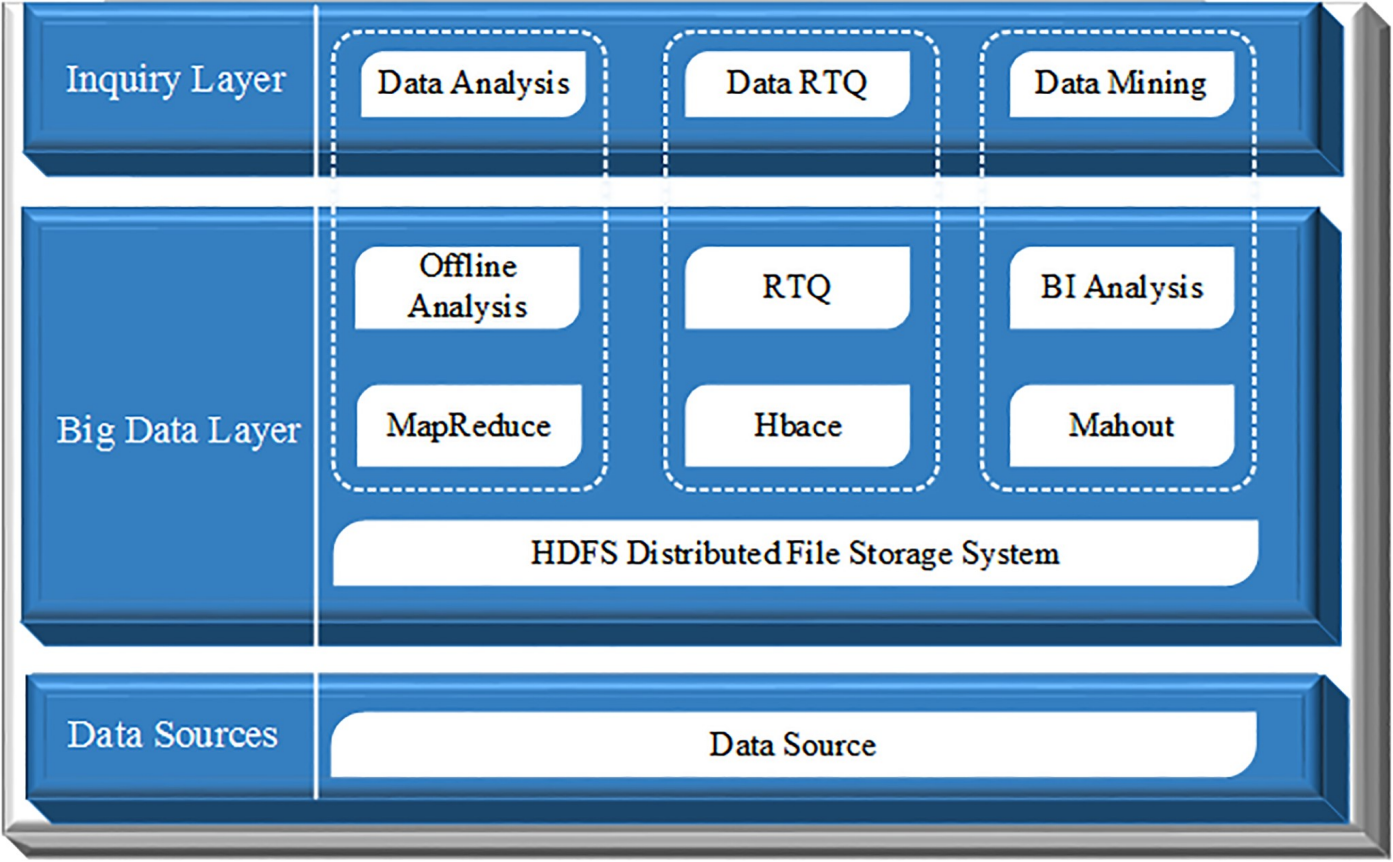
Overall framework of Hadoop platform.

Hadoop is an open-source and distributed computing platform with three layers. The first layer is the underlying data source, composing of a large amount of data generated by each user. The middle layer, as the core layer of Hadoop, is also called big data layer, which includes the two cores of the Hadoop ecosystem: Hadoop Distributed File System (HDFS) and MapReduce processing system. In addition, the big data layer also contains the distributed column database Hbace for real-time query, and the big data algorithm library Mahout [13]. The third layer is the Inquiry layer which can analyze, query, and mine data. The overall framework of Hadoop platform is shown in Fig 2.

#### Hadoop Distributed File System

**Fig 3:**
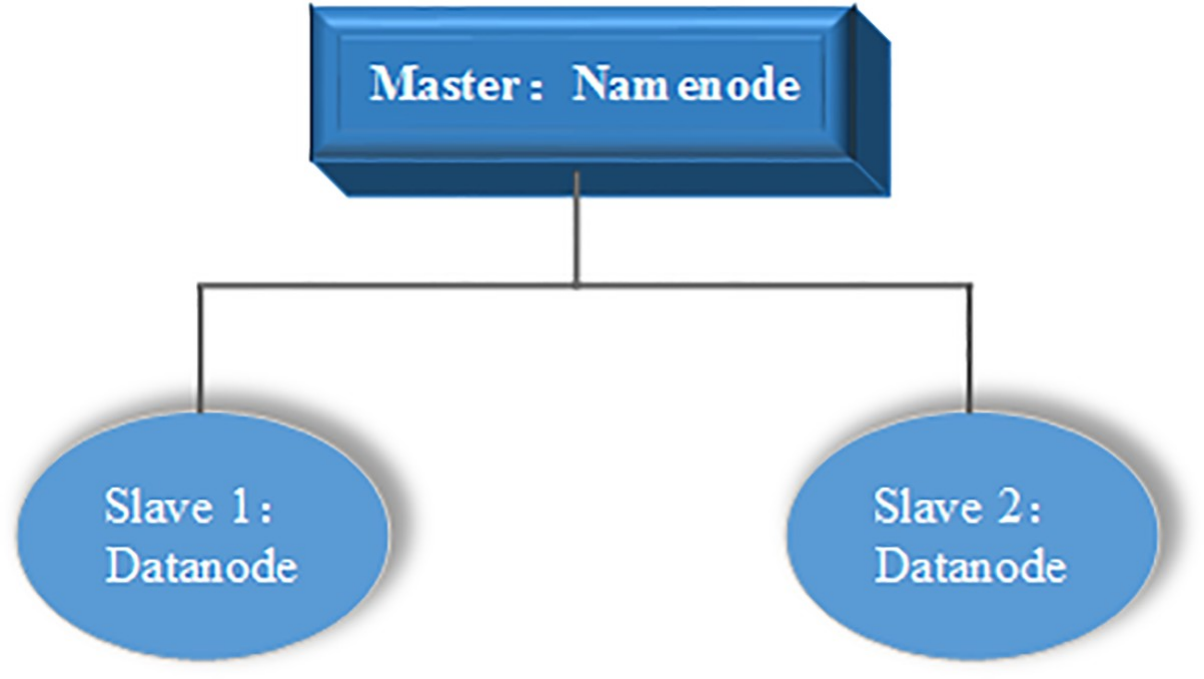
HDFS frame structure.

As shown in Fig 3, HDFS adopts master/ slave architecture. Each cluster contains a name node and several data storage nodes. The name node is responsible for generating data directory and managing users' access to data files while the data storage node is used to store data files.

#### MapReduce

**Fig 4:**
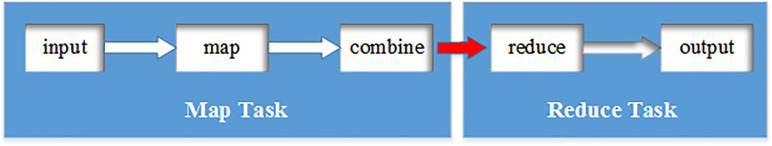
MapReduce data flow diagram.

In Hadoop system, the operation can be divided into two stages, Map and Reduce respectively [14]. During the Map stage, a set of intermediate key-value pairs based on the input key-value is generated. Then, MapReduce framework collects the generated intermediate key-value pairs and distributes them to Reduce. Reduce is used to process intermediate key-value pairs and related sets. Finally, these key-value pairs are merged to obtain results, as shown in Fig 4.

### Random forest classification algorithm

Random forest algorithm is an integrated learning method, the framework of which is a bagging algorithm one. A decision tree classifier is adopted for base classifier, so as to classify both continuous variables and discrete variables [15]. Random forest algorithm is highly capable of very precise generalization, proposes no requirements for training data attributes, and can meet the requirements of high parallel operation of big data processing. It can be well integrated with MapReduce processing system of the Hadoop platform. The electric submersible screw pump data set mainly has two features: numerical variables and category variables. Therefore, random forest algorithm is applied to diagnose and analyze submersible screw pump faults.

#### Bagging algorithm

Bagging sampling algorithm refers to multiple rounds of random sampling with replacement of the overall data. Each round can extract various sets of data. The probability of each group of data selected is line with the uniform probability distribution. After multiple rounds of data sampling, multiple training sets are obtained, and each training set generates a base classifier. All the base classifiers classify and vote on the data to be tested respectively, and finally determine the data to be tested as the highest category of votes [16]. The process of bagging algorithm is shown as follows:

1. Divide the overall data into training data and test data;
2. Randomly take n sets of sample data from the training data to form a training sample set;
3. Repeat step (1) k times to get k training sample sets. The training sample sets are independent of each other, and different training sample sets may have duplicate elements;
4. In terms of K training sample sets, train K models, which are determined by specific problems;
5. The final result is produced by voting carried by the results of K models.

#### Basic principles and algorithm of decision tree

**Fig 5:**
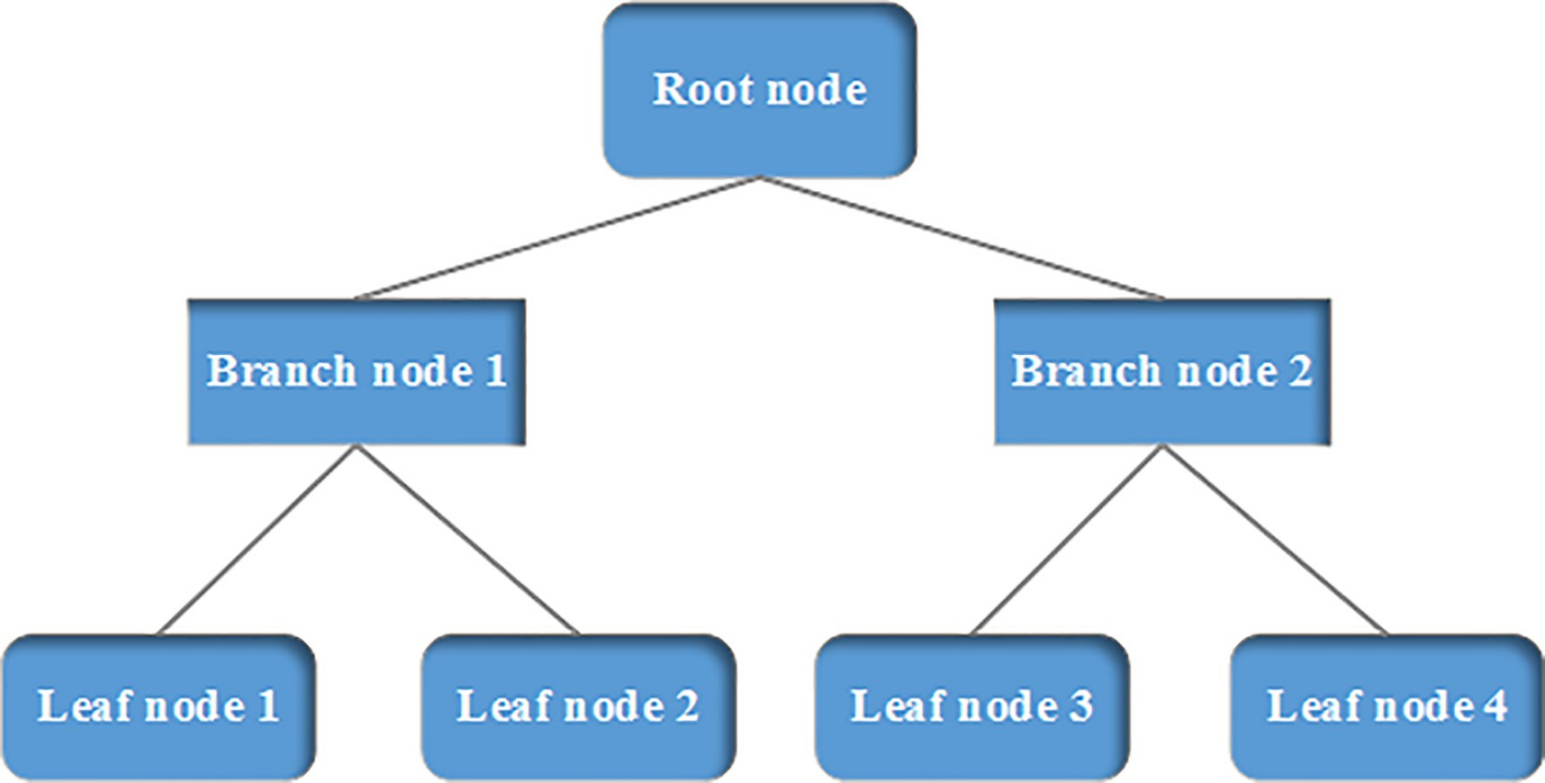
Decision tree classification.

Decision tree has an inverted tree structure. The nodes include root node, branch node and leaf node. The root node represents a type of test and is located at the top: the test is conducted by using attribute 1. Different branch nodes represent varied test results. Then, attribute 2 is used to get the leaf node. The leaf nodes store the classification label value, representing any possible classification results [17]. The decision tree algorithm is used to classify the unknown samples. The classification process is shown in Fig 5 as follows.

Three common methods are usually used for building a decision tree: ID3 algorithm, C4.5 algorithm and cart algorithm. The main difference lies in the evaluation methods used for different attributes of original data.

(1) ID3 algorithm

The ID3 algorithm uses the information entropy of different attributes as the judgment criterion when selecting data feature attributes. To this end, one specific attribute for node splitting is selected [18]. Entropy is an indicator for the purity of sample set. The calculation formula of information entropy is shown as follows:

$$Entrop\left(S\right)=‐\sum_{i=1}^{m}p_{i}\log_{2}\left(p_{i}\right)$$ (1)

Where, *m* represents the total number of sample attributes. *p_i_* means the probability of obtaining *NO*.*i* attribute; *Entrop(S)* refers to the information entropy when attribute is not taken into consideration. If the value of *Entrop(S)* decreases, the purity of the data sample will become higher.

However, ID3 can only discrete feature variable attributes rather than handle continuous variable feature attributes. At the same time, ID3 generally inclines to select feature attributes with more attribute values, which reduces the classification accuracy.

(2) C4.5 algorithm

Information gain is used to measure the importance of an attribute in the data set [19]. For example, the information gain of attribute *A* is as follows:

$$Gain\left(S,A\right)=Entrop\left(S\right)-\sum_{v\in \mathrm{Value}\left(A\right)}\frac{\left|S_{V}\right|}{S}Entrop\left(S_{V}\right)$$ (2)

Where, *Value(A)* refers to the set of values of attribute *A*. *V* is a certain attribute value of *A*. *S_V_* represents the sample set with a value of *V* in *S*. |*S_V_*| means the amount of samples contained in *S_V_*. The larger the value of information gain *Gain*(*S,A*) is, the more important attribute *A* in the dataset becomes. Samples should be divided according to attribute *A*.

In feature selection process, C4.5 algorithm uses information gain rate as the standard indicator to circumvent the problem arising from ID3 algorithm [20]. The information gain rate of attribute *A* is expressed as:

$$Gain\_ratio\left(A\right)=Gain\left(S,A\right)/\left(-\sum_{v\in \mathrm{Value}\left(A\right)}\frac{\left|S_{V}\right|}{S}\log_{2}\frac{\left|S_{V}\right|}{\left|S\right|}\right)$$ (3)

The feature attributes with high information gain rate is influential on the data set, so the feature attributes with high information gain rate are preferred when splitting the decision tree.

On the one hand, C4.5 algorithm effectively solves the problems of ID3 algorithm, enhances the rationality of decision tree and improves the classification accuracy. However, on the other hand, C4.5 algorithm also has certain defects: the algorithm has to repeatedly calculate and traverse the data, and store all the data sets. Hence, more time is needed for calculating the algorithm and more space is needed.

(3) Cart algorithm

Cart algorithm uses the minimum Gini index criterion in the node splitting of decision tree and adopts Gini index to reflect the purity of the sample [21]. Gini index is similar to information entropy, but it requires obviously less calculation than information entropy. Cart tree adopts node dichotomy to remove ID3’s defect which tends to select more variables of categories. The calculation formula is as follows:

$$Gini\left(S\right)=\sum_{i=1}^{m}p_{i}(1-p_{i})=1-\sum_{i=1}^{m}p_{i}^{2}$$ (4)

In nature, Gini index is to randomly take two groups of data from the sample set S. These two groups of data are within the probability of the same category. Therefore, the smaller the Gini coefficient is, the higher the purity of the sample set is. If *A* is a characteristic attribute of the sample set S, then the Gini coefficient of characteristic attribute *A* is as follows:

$$Gini\left(S,A\right)=\sum_{v\in \mathrm{Value}\left(A\right)}\frac{\left|S_{V}\right|}{S}Gini\left(S_{V}\right)$$ (5)

## Fault diagnosis model of submersible screw pump based on random forest algorithm

### Fault diagnosis process of submersible screw pump

The fault diagnosis for submersible screw pump based on random forest algorithm depends on big data processing platform. The specific process is as follows:

1. Build a Hadoop big data processing platform and establish a distributed file processing system of HDFS and MapReduce processing system;
2. Cleanse the data collected in the oil field, remove the data noise and data that is not related to mining, and fill in the missing data;
3. Evaluate the importance of characteristic attributes of all relevant data to submersible screw pumps and sort out data based on their rank of importance;
4. Divide data into a training set and a test set. In the training set, the Bagging sampling method is adopted to establish a sample library, and then the CART method is used to build a decision tree to form a random forest. The test set aims to test the accuracy of the random forest model to diagnose faults;
5. Input the data to be logged, call the random forest model to judge, and finally output the diagnosis results.

### Data characteristics analysis of submersible screw pump

After the field investigation, the following working parameters are mainly collected for submersible screw pump wells in the oilfield: current, speed, voltage, active power, power factor, reactive power, submergence, pump hanging depth, liquid production, oil production, oil pressure and casing pressure. Also, the failure category of screw pump is written down. In addition, well fluid parameters are included, such as sand and wax content. The basic parameters of submersible screw pump wells cover pump type and well number. All the parameters are shown in Table 1.

**Table 1:** Working parameters of submersible screw pump.

Serial number	Characteristic attributes	Letter code	Unit	Variable type	Remarks
**1**	Electric current	I	A	Continuous	Real time monitoring
**2**	Speed	n	r/min	Continuous	Real time monitoring
**3**	Voltage	U	V	Continuous	Real time monitoring
**4**	Active power	Pa	kW	Continuous	Calculated
**5**	Power factor	Pf	-	Continuous	Calculated
**6**	Reactive power	Pr	Kw	Continuous	Calculated
**7**	Submergence	Sub	m	Continuous	Capillary test
**8**	Pump hanging depth	Dep	m	Continuous	Measure
**9**	Moving liquid level	Dy	m	Continuous	Calculated
**10**	Liquid production	Yl	t/d	Continuous	Daily output
**11**	Oil production	Yo	t/d	Continuous	Daily output
**12**	Water content	Yw	t/d	Continuous	Daily output
**13**	Oil pressure	Pc	MPa	Continuous	Once a day
**14**	Casing pressure	Pt	MPa	Continuous	Once a day
**15**	Sand bearing	Sand	-	Categorical	Value 0^a^ or 1^b^
**16**	Waxy	Wax	-	Categorical	Value 0 or 1
**17**	Pump type	Type	-	Categorical	-
**18**	Well No.	Well	-	Categorical	-

^**a**^**0 indicates that the sand and wax contents are relatively low.**

^**b**^**1 indicates that the sand and wax contents are relatively high.**

The random forest algorithm is used to analyze the faults and the data. The specific process is as follows:

1. Among several sets of feature attributes with high correlation, one set of input data are reserved as a fault diagnosis model. Among current, voltage, active power, power factor and reactive power, active power is selected as the model input data; among submergence, pumping depth and dynamic liquid level, submergence is selected as the model input data; among fluid production, oil production and water content, liquid production is selected as the input data of the model.
2. The active power is calculated based on the current and voltage monitored in real time. To calculate the active power, the researcher takes the data of a period before the failure and analyzes the data of this period of time. The data of this period of time can be regarded as a vector. The data mainly have two statistical characteristics, the central measurement of the data and the measurement of the dispersion degree of the data. These two characteristics are used in processing the power vector to characterize this data with two continuous variables of mean and variance. In this way, the power data is processed into two continuous variables. The mean value of active power is represented by "Mean" and the variance, by "Sd".
3. Finally, six continuous variables, including mean active power, active power variance, submergence, liquid production, oil pressure and casing pressure, and four categorical variables, including sand, wax, pump type, and well number, are chosen to be input in the random forest model, to train a random forest model, and analyze faults on submersible screw pumps.

### Data pre-processing

As the data are originated from different systems, there are certain differences in data format and integrity. In order to facilitate data mining, it is necessary to clean the data.

Standardization of names

Hashtag information is unified. There are two formats of the original well number, one is the combination of Chinese characters and numbers, the other is the combination of letters and numbers.

(2) Time field normalization

The recording intervals are different for each working parameter data of submersible screw pump in the oil field. Some parameters are monitored in real time, such as, current, active power, voltage, power factor, and reactive power, while some parameters are measured once a day, such as oil pressure, casing pressure, and dynamic liquid level. The time format is normalized during data cleansing.

(3) Normalization of relevant data units

The units for the data from different oil fields are unified and labelled.

(4) Data missing value processing

Due to machine or human reasons, the recorded data may have missing values. Combined with the actual situation of the monitoring parameters of submersible screw pump wells, the missing values will be filled or deleted.

After data cleaning, the data from different oil fields and departments are merged. Based on the fault category, the current, voltage, active power, power factor, reactive power, oil pressure, casing pressure, dynamic liquid level and other working parameter data of the submersible screw pump well are combined into one list when different faults occur, a total of 132 groups of submersible screw pump failure data are sorted out, 90 groups of which are randomly selected as training data, 42 groups are selected as test data, and stored in the HDFS distributed storage system.

### Descriptive files generated

The parameters of the random forest model are set according to the input data set, including the size of the training set, the type of data variables in the training set, and the characteristics of the training set.

The training data for the fault diagnosis model on submersible screw pump mainly include the following 10 parameters: six continuous variable parameters (active power mean, active power variance, submergence, liquid production, oil pressure, casing pressure) and four categorical variable parameters, including sand, wax, pump type and well number. Part of the data is shown in Table 2.

**Table 2:** Part of the data of the fault diagnosis model.

Serial number	Well No.	Active power mean/kW	Active power variance	Sub/m	Pc/MPa	Pt/MPa	Yl/(t/d)	Pump type	Wax	Sand	Working condition category
**1**	B2—312-53	1.9685	0.1376	13	0.06	3.30	10	200	0	1	0
**2**	B3-D5-P51	1.5417	0.0413	20	0.21	1.20	7	200	1	0	0
**3**	B3-D3-P51	1.0728	0.1151	515	0.12	1.20	2	200	0	1	0
**. . .**	. . .	. . .	. . .	. . .	. . .	. . .	. . .	. . .	. . .	. . .	. . .
**23**	B2—D3-55	1.1106	0.0874	876	0.32	1.20	61	200	1	0	1
**24**	B3—D5-P32	0.8512	0.4888	495	0.21	3.10	79	200	1	1	1
**25**	B2—D3-425	1.3079	0.1384	480	0.11	0.80	63	200	0	1	1
**. . .**	. . .	. . .	. . .	. . .	. . .	. . .	. . .	. . .	. . .	. . .	. . .
**45**	B2—D3-455	1.3998	0.3178	926	0.12	1.52	54	200	1	0	2
**46**	B3—D5-P59	1.5638	0.0374	1048	0.31	3.91	44	200	1	0	2
**47**	B3—D5-P57	1.5598	0.0102	441	0.34	2.22	57	200	1	1	2
**. . .**	. . .	. . .	. . .	. . .	. . .	. . .	. . .	. . .	. . .	. . .	. . .
**67**	B2—D3-P69	1.6729	0.0089	515	0.11	1.71	69	200	0	0	3
**68**	B2—20-455	1.4321	0.0243	783	0.17	2.60	48	100	0	1	3
**69**	B2—D4-P56	2.0112	0.1442	352	0.21	2.71	75	200	0	1	3
**. . .**	. . .	. . .	. . .	. . .	. . .	. . .	. . .	. . .	. . .	. . .	. . .
**89**	B2—342-P36	2.0206	0.0896	467	0.02	2.70	2	100	0	0	4
**90**	B3—20-P51	1.7439	0.0317	777	0.05	3.20	3	200	1	0	4
**91**	B2—D3-SP65	2.0365	0.0333	1038	0.01	2.30	1	200	0	0	4
**. . .**	. . .	. . .	. . .	. . .	. . .	. . .	. . .	. . .	. . .	. . .	. . .
**130**	ZF90-52	2.0943	0.0329	343	0.11	3.70	48	100	0	0	5
**131**	ZF81-47	2.4628	0.5735	325	0.30	1.60	56	200	0	1	5
**132**	B2-342-P37	5.1565	7.3457	798	0.30	1.40	66	100	0	0	5

**Fig 6:**
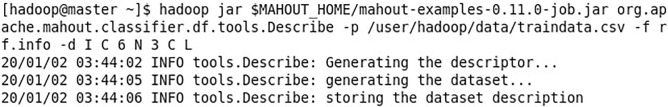
Descriptive documents.

According to the type of input data, a descriptive file is generated. The description file is used to input the category attributes of the training data into the computer and provide the information about whether to participate in modeling, and other information. The first column of the training data of the submersible screw pump fault diagnosis model is the serial number column, which is not used in the modeling, and is represented by "I"; the second column and 9–11 columns of the training data refer to the categorical variables, and are represented by "C"; Column 12 is the category label, namely, the screw pump fault category code, "0–5" represents six working status of "pump emptying, pump leakage, wax plugging, pipe leakage, flexible shaft failure, normal operating conditions" in the submersible screw pump, which are indicated by "L" in the program. Fig 6 shows the procedure and process for generating descriptive files. The generated descriptive file "rf.info" is stored in HDFS and is put aside before the model is built.

### Fault diagnosis model of submersible screw pump

#### Construction of random forest model

**Fig 7:**
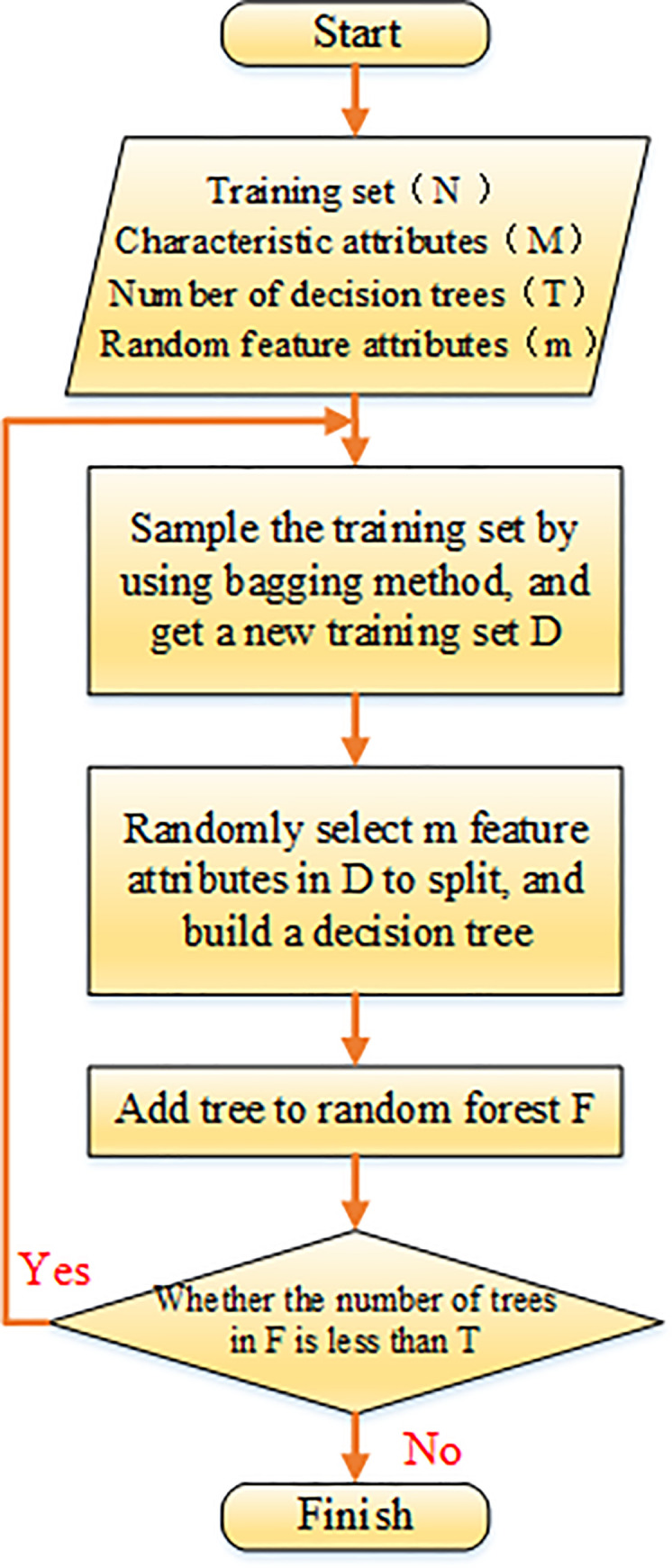
Flow chart of generating random forest model.

**Fig 8:**
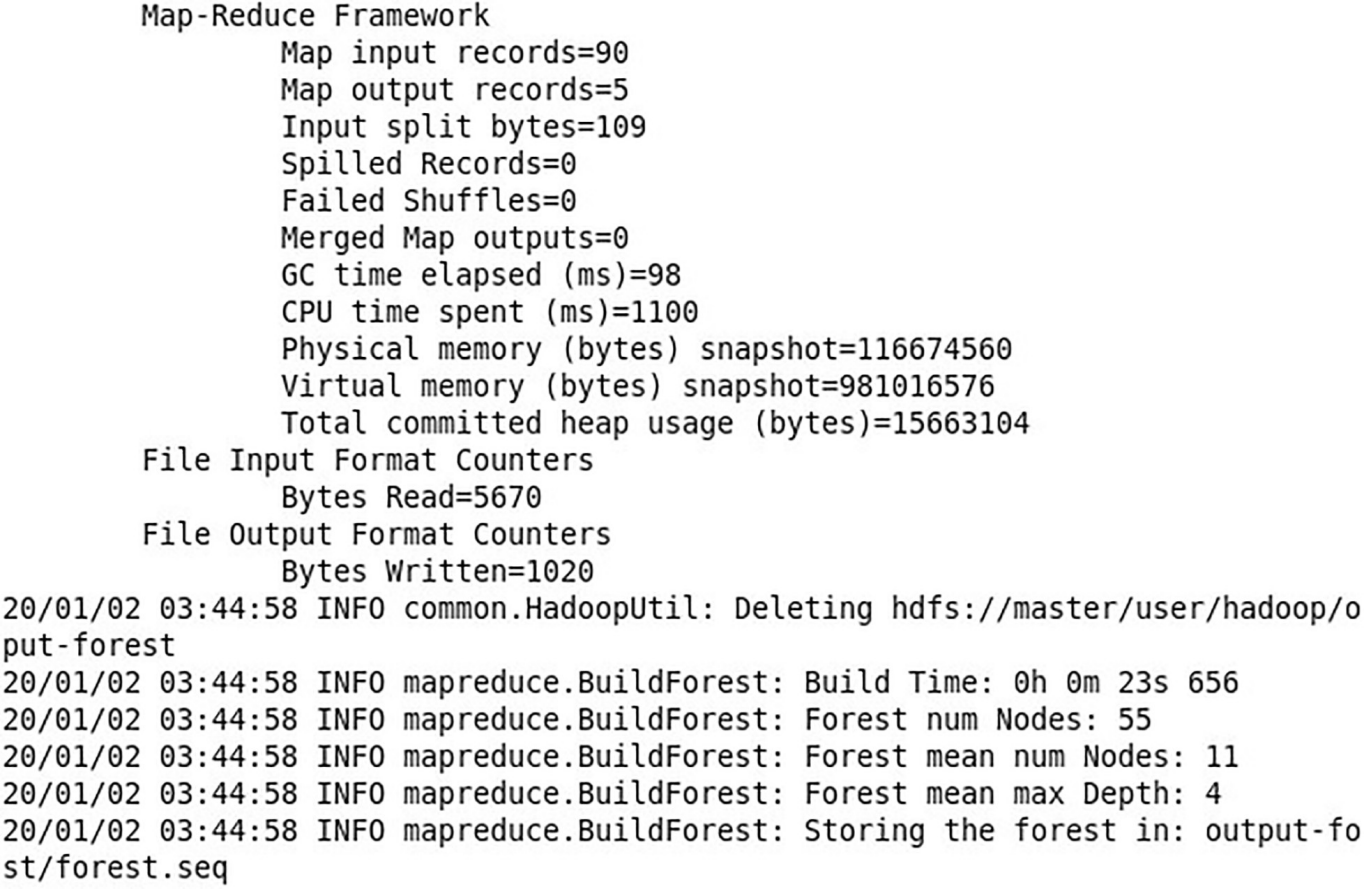
Random forest model.

The original training set is collected using bagging sampling. The new training set D_t is sampled, and the sampling is performed for T times; and m feature attributes are selected from the training set D_t to split the decision tree for establishing a decision tree. Each training set generates a decision tree, and finally T decision trees are generated and converted into a random forest model. The modeling process and the modeling results are shown in in Figs 7 and 8 respectively.

Based on the results, there are 90 input records, more specifically, 90 sets of training data; and 5 output records, a random forest built based on 5 decision trees. The decision tree is split up for 4 times, and the generated random forest model is stored in the forest.seq file in the output-forest folder.

#### Evaluation of the random forest model

**Fig 9:**
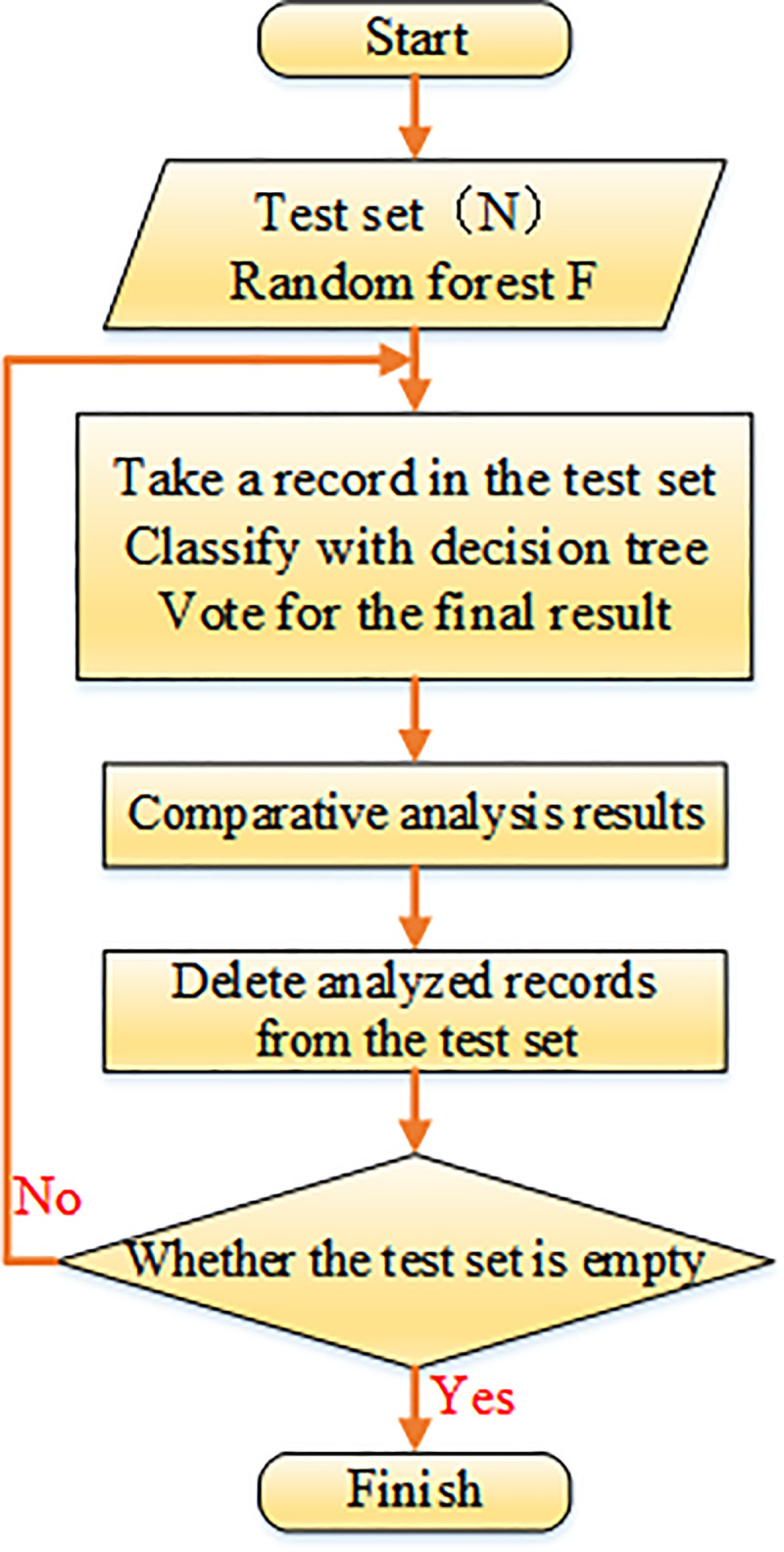
Flow of testing random forest model.

**Fig 10:**
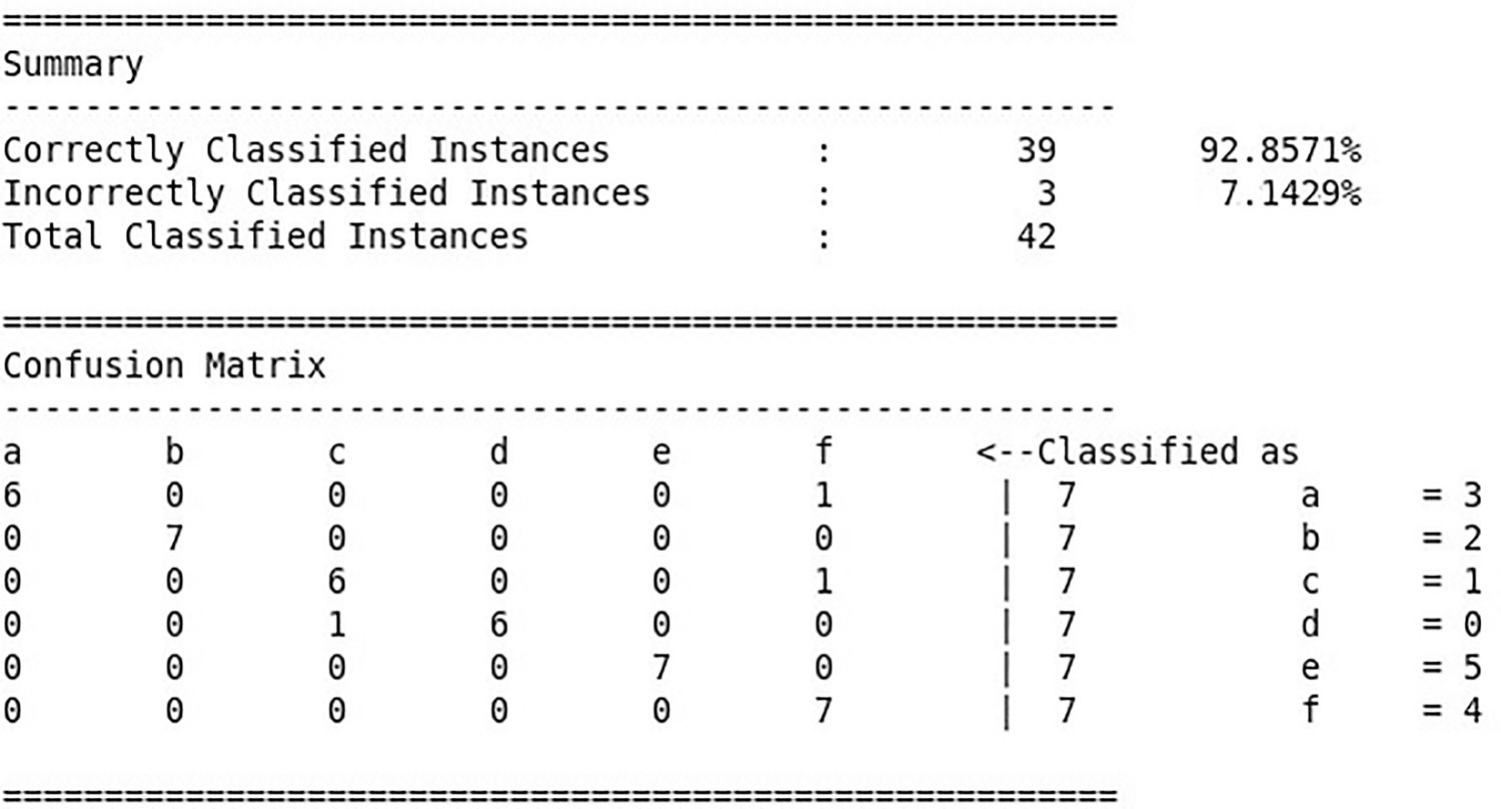
Test results of random forest model.

The test data are used to evaluate the random forest model generated above and analyze the accuracy rate. The process is shown in Fig 9. The test data is input into the random forest fault diagnosis model, to obtain the result shown in Fig 10. The result includes the test accuracy rate and the confusion matrix, which is obtained by using the test set in the random forest model.

From the results, it can be seen that the random forest model categorizes 42 sets of test data. Based on the reference to the fault category code of submersible screw pump, wax plugs and flexible shaft faults are easier to distinguish, and other fault categories are all misjudged. The submersible screw pump fault diagnosis model correctly classifies 39 sets of data, while errors occur when the model classifies 3 sets of data classification errors. The accuracy rate is about 92.86%, indicating that the random forest-based submersible screw pump fault diagnosis model works well and can be applied to reality.

In order to verify the random forest fault diagnosis method, the researchers used both BP neural network and probabilistic neural network for fault diagnosis based on the above data. The fault diagnosis method based on BP neural network and probabilistic neural network is compared with the random forest fault diagnosis method. The test accuracy rates of the latter two diagnostic methods are 86% and 90.5%, respectively. The accuracy of the random forest-based algorithm is improved by 6.86 and 2.36 percentage compared with the other two methods.

### Comparative analysis of fault diagnosis methods

Previous scholars mainly studied the fault diagnosis method of submersible screw pump based on neural network. However, this paper proposes the fault diagnosis method of submersible screw pump based on random forest algorithm. Therefore, for these two methods, a comparative analysis is carried out on the parameters, algorithms and scope of application.

#### Comparative analysis of parameters

The two fault diagnosis methods are based on the operating data and various parameters of the submersible screw pump to identify the working conditions, but the specific parameters are different. The diagnosis method based on neural network uses five parameters of active power, liquid production, oil pressure, casing pressure, and dynamic liquid surface depth as the basis for fault diagnosis; The diagnosis method based on random forest includes the following 10 parameters: six continuous variable parameters (active power mean, active power variance, submergence, liquid production, oil pressure, casing pressure) and four categorical variable parameters, including sand, wax, pump type and well number.

#### Comparative analysis of algorithm

The neural network-based diagnosis method takes active power as the core parameter, performs wavelet packet decomposition and reconstruction, and obtains the energy information contained in each frequency band. Under different working conditions, the energy contained in the same frequency band is different, so as to determine the fault category of the screw pump. The fault diagnosis method based on random forest processes the data statistically, analyzes the potential correlation between parameter data and fault categories, uses bagging algorithm to sample the original data, and establishes a decision tree classifier to form a random forest model.

#### Comparative analysis of scope of application

At present, the long-term monitoring parameters that can be directly obtained in screw pump wells mainly include voltage, current, production, oil pressure, casing pressure, dynamic liquid surface depth, etc. The fault diagnosis method based on neural network is suitable for this situation. However, with the continuous development of smart oil fields and big data, the database will be more robust, and the use of big data platforms to process data will be faster and more efficient. In this case, the fault diagnosis method of submersible screw pump based on random forest is more applicable and the analysis result is more accurate.

## Conclusion

1. Given the difficulty in diagnosing faults and low accuracy of current fault diagnosis methods of submersible screw pumps, this paper proposes a fault diagnosis method of submersible screw pump based on random forest. Six continuous variables and four classified variables of the production system of submersible screw pump are used to analyze and judge the fault in the wells. The parameters are fully used and the accuracy is high.
2. Based on the establishment of Hadoop big data processing platform, HDFS distributed file storage system and MapReduce parallel processing system are established to store and process the data for submersible screw pump.
3. Bagging algorithm is used to take samples from the training set data and establish the sample database. The cart method is used to establish the decision tree and forms the random forest model. The accuracy rate of the model is 92.86%, which proves the fault diagnosis methods can be applied to the fault diagnosis of submersible screw pumps.

## Supporting information

S1 File (ZIP)

S1 Table Working parameters of submersible screw pump. (XLS)

S2 Table Part of the data of the fault diagnosis model. (XLS)
